# Pathogenic variants among females with breast cancer and a non-breast cancer reveal opportunities for cancer interception

**DOI:** 10.1007/s10549-023-06870-x

**Published:** 2023-03-01

**Authors:** Brittany L. Bychkovsky, Min-Tzu Lo, Amal Yussuf, Carrie Horton, Parichehr Hemyari, Holly LaDuca, Judy E. Garber, Rochelle Scheib, Huma Q. Rana

**Affiliations:** 1 Department of Medical Oncology, Dana-Farber Cancer Institute, 450 Brookline Ave, Boston, MA 02215 USA; 2 Breast Oncology Program, Dana-Farber Brigham Cancer Center, Boston, MA USA; 3 Division of Cancer Genetics and Prevention, Dana-Farber Cancer Institute, Boston, MA USA; 4 Harvard Medical School, Boston, MA USA; 5 Ambry Genetics, Aliso Viejo, CA USA

**Keywords:** Multiple primary cancers, Breast cancer, Genetics, Germline, Pathogenic variants, Cancer interception, Survivorship

## Abstract

**Purpose:**

Herein, we report the frequency and distribution of germline pathogenic variants (PVs) among females with breast cancer (BC) and at least one other non-BC who underwent multi-gene panel testing (MGPT). Among females with PVs diagnosed first with BC or ovarian cancer (OC), we sought to enumerate the frequency of subsequent PV-associated cancers.

**Methods:**

Females with BC and cancer of ≥ 1 other site (multiple primary cancers, MPC) who underwent MGPT through Ambry Genetics from March 2012 to December 2016 were included if they had testing of at least 21 genes of interest **(***ATM, BARD1, BRCA1, BRCA2, BRIP1, CDH1, CHEK2, EPCAM, MLH1, MSH2, MSH6, MUTYH, NBN, NF1, PALB2, PMS2, PTEN, RAD51C, RAD51D, STK11,* and *TP53*)*.* Phenotypic data were abstracted from test requisition forms and clinical notes.

**Results:**

Of 6,617 evaluable patients, most were White (70.8%) and median age at first cancer, second cancer, and MGPT was 49 (interquartile range [IQR]: 18), 59 (IQR: 16), and 63 (IQR: 16) years, respectively. PVs were found among 14.1% (932/6617) of the overall cohort and in 16.4% (440/2687) of females who were diagnosed first with BC. Among those, 55.2% (243/440) had an actionable PV associated with a subsequent cancer diagnosis including 150 OCs. Of the 2443 females with breast and ovarian cancer, few (*n* = 97, 9.5%) were diagnosed first with OC, limiting our analysis.

**Conclusions:**

Females with MPC, including BC, have a high frequency of germline PVs (14.1%). These data delineate the opportunities for intercepting subsequent cancers associated with genetic risk among females diagnosed first with BC.

**Supplementary Information:**

The online version contains supplementary material available at 10.1007/s10549-023-06870-x.

## Introduction

Multiple primary cancers (MPCs) and, notably, subsequent cancer diagnoses among breast cancer survivors are multifactorial. While some are due to receipt of chemotherapy or radiation or exposure to a common carcinogen, others can be attributed to a genetic risk [1]. In prior work, we found that patients with MPCs who underwent germline genetic testing had a high rate of pathogenic variants (PVs) (> 10%) regardless of age at their second cancer diagnosis (< 50, 50–70 or > 70) [2]. Patients with breast cancer (BC) who develop a second BC are more likely to have PVs in germline BC genes (*ATM, BRCA1, BRCA2, CHEK2, CDH1, NBN, NF1, PABL2, PTEN,* and *TP53*) than those with one BC [3]. Additionally, patients with BC and any other primary cancer diagnosis are more likely to have a PV in a variety of cancer genes than those with a BC only (8.5% vs. 4.9%) [4]. Data remain limited on the frequency of PVs among BC survivors who develop a subsequent cancer diagnosis.

Germline genetic testing of female patients with BC is often limited to those with a strong cancer family history, early age at diagnosis, and specific tumor subtypes [5]. Family history of *BRCA*-associated cancers (breast, ovarian, pancreatic, and prostate) and risk models that prompt a referral for germline genetic testing (BOADICEA, BRCAPRO, Penn II, and Myriad) emphasize *BRCA*-related cancer risk [6]. Evidence supporting targeted therapies for early BC has led to calls for more liberal germline genetic testing of patients with BC at diagnosis to inform surgical and therapeutic options [7]. Moreover, as BC care and survivorship continue to improve, it is increasingly important to counsel BC patients on future cancer risks, including the risk of a second BC and the risk of a subsequent non-breast primary cancer.

Likewise, while genetic testing has been recommended for all patients with ovarian cancer (OC) in the National Comprehensive Cancer Network Clinical Practice Guidelines in Oncology (NCCN Guidelines®) since 2010, there has been low compliance with this guideline [8]. Numerous quality improvement initiatives have been undertaken to address this testing gap [9, 10]. As survivorship in OC improves, owing in part to targeted and maintenance therapy [11, 12], subsequent cancers may emerge as an important issue for this patient population.

The primary aim of this study was to delineate the frequency and distribution of germline PVs/likely PVs among females with BC and a primary cancer of another non-breast site who underwent multi-gene panel testing (MGPT). We examined the frequency and distribution of PVs by combinations of cancers. We report PV frequencies by the order of cancer diagnosis (referred to as diagnostic order) for BC and OC. Next, we sought to elucidate the opportunities for reducing cancer morbidity and mortality through quantifying the frequency of subsequent cancers associated with patients’ PVs. We limited our analysis to subsequent cancers that are known to be associated with germline PVs whose outcomes are influenced by early detection or interception (e.g., BC and colorectal cancers), or which may be prevented by risk-reducing procedures (breast, colorectal, endometrial, and ovarian cancers). Among the subset of females with germline PVs who were diagnosed first with BC and separately with OC, we quantified the opportunities for cancer interception [13].

## Methods

### Subjects

A retrospective cross-sectional study of reported cancer history and genetic test results was conducted of tested individuals through a diagnostic testing laboratory (Ambry Genetics, Aliso Viejo, CA). Females were included if they had a personal diagnosis of BC and ≥ 1 non-BC diagnosis (excluding nonmelanoma skin cancers) and had MGPT between March 2012 and December 2016, inclusive of the following 21 genes: *ATM, BARD1, BRCA1, BRCA2, BRIP1, CDH1, CHEK2, EPCAM, MLH1, MSH2, MSH6, MUTYH, NBN, NF1, PALB2, PMS2, PTEN, RAD51C, RAD51D, STK11,* and *TP53.* Concurrent cancers at one site were counted once only (e.g., two colorectal cancers at the same age counted as one cancer). Patients with multiple PVs were enumerated and excluded from the analytic cohort and are the subject of a separate analysis.

Variant interpretation was performed based on the American College of Medical Genetics and Genomics and the Association of Medical Pathologists guidelines [14, 15]. PVs and likely PVs were both denoted as PVs in this study. The lower-risk variant, *CHEK2* p.I157T, was distinguished from other PVs in *CHEK2* and included in overall counts. Biallelic *MUTYH* was considered a PV and monoallelic *MUTYH* was not. Per NCCN Guidelines® for Breast/Ovarian Genetic/Familial High-Risk Assessment V1.2022 [16], we categorized *BRCA1, BRCA2, CDH1, PALB2, PTEN*, and *TP53* as high-risk BC genes; *ATM, BARD1, CHEK2* (excluding *CHEK2* p.I157T), *NBN* 657del, and *NF1* as moderate-risk BC genes; *CHEK2* I157T, *RAD51C*, and *RAD51D* as “other BC” genes. The DNA Mismatch Repair (MMR) genes included the following: 3’ deletions in *EPCAM, MLH1, MSH2, MSH6,* and *PMS2.* We limited our analysis to these 21 genes because they are commonly tested for, often have distinct screening or risk-reducing strategies for cancer interception and most have been associated with more than one cancer type. This study was exempt from review by the Western Institutional Review Board.

Clinician-completed requisition forms and clinical documentation (pedigrees and chart notes) were abstracted for clinical characteristics including cancer history and demographics (sex, age at testing and at each tumor diagnosis).

### Data analysis

Descriptive statistics are summarized as medians (interquartile range [IQR]) for continuous and proportions for categorical patient characteristics. T-tests were used to examine differences in ages at panel testing, first and second primary cancer diagnosis. For diagnostic order of BC, we performed chi-squared tests. We also performed chi-squared test for trend to examine whether there was a significant increasing/decreasing trend for the PV frequency by the diagnostic order for OC. All statistical tests were two-sided, and a *P* value of < 0.05 was considered statistically significant. A continuity correction was used for the 95% confidence interval (*CI*) when the numerator was < 10. All analyses were conducted with R v.3.3.3.

## Results

### Cohort characteristics

**Fig. 1:**
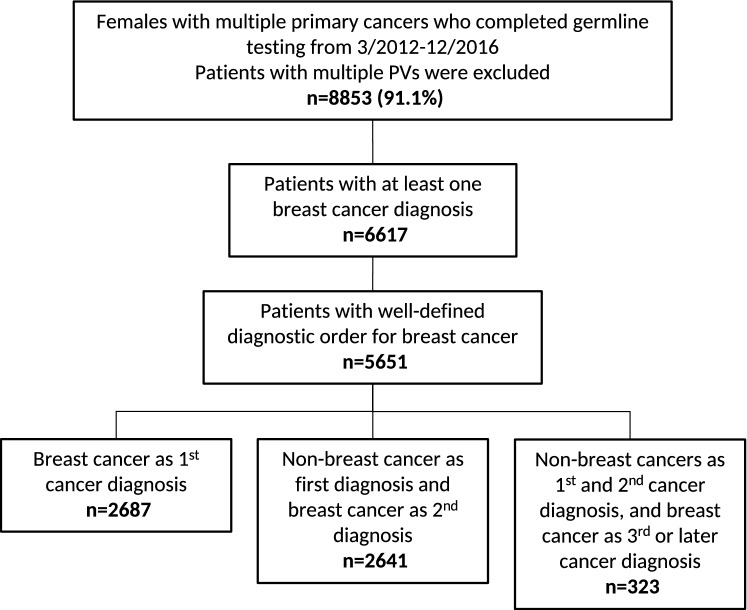
Strobe diagram**. **Legend: PV(s), pathogenic, or likely pathogenic variant(s)

From March 2012 to December 2016, 9,820 patients with multiple primaries underwent germline multi-gene panel testing at Ambry Genetics that included all 21 genes of interest as previously described (Fig. 1) [7]. Patients with multiple PVs (*n* = 96) were (excluded from further analysis. Among the 9,714 remaining patients with MPCs (2–6 primary tumors), 6,617 (68.1%) had female BC. Among the analytic cohort (6617), most were White (70.8%) and had two primary tumors (84.5%). The median age at first cancer diagnosis was 49 (*IQR* 18), median age at second cancer diagnosis was 59 (*IQR* 16), and median age at testing was 63 years (*IQR* 16, Table 1).

There were 1,007 (15.2%) patients with bilateral BCs: 25.6% (*n* = 258) were diagnosed with synchronous bilateral BCs and the remaining 74.4% (*n* = 749) were asynchronous. Hormone receptor data were missing from 45.5% of BC cases. Among the 3,609 females with available hormone receptor subtype, 79.3% had estrogen-receptor-positive (ER +) and/or progesterone-receptor-positive (PR +) disease. Among the 3,525 females with hormone receptor subtype and human epidermal growth factor receptor 2 (HER2) subtype also available, 15.0% had triple negative breast cancer. Notably, 2443 (36.9%) also had diagnosis of OC, though only 1022 (18.3%) had only BC and OV among the 5594 patients with two cancer diagnoses only.

**Table 1:** Characteristics of analytic cohort for females with breast cancer and multiple primary tumors stratified by age, ethnicity/ancestry, breast cancer subtype, and diagnosis of bilateral breast cancer

	Female breast cancer (*N* = 6617)	Breast cancer as 1^st^ diagnosis (*N* = 2687)
	Number (%)	Number (%)
Age at testing (years)		
< 30	13 (0.2%)	3 (0.1%)
30–49	835 (12.6%)	217 (8.1%)
50–69	3921 (59.3%)	1580 (58.8%)
70 +	1848 (27.9%)	887 (33.0%)
Range	22–90 +	25–90 +
Mean (*SD*)	62.8 (11.4)	64.8 (10.8)
Median (*IQR*)	63 (16)	65 (14)
Ethnicity		
Caucasian	4687 (70.8%)	1915 (71.3%)
Ashkenazi Jewish	452 (6.8%)	178 (6.6%)
African American/Black	295 (4.5%)	135 (5.0%)
Hispanic	217 (3.3%)	102 (3.8%)
Asian	188 (2.8%)	71 (2.6%)
Mixed Ethnicity	337 (5.1%)	121 (4.5%)
Other	47 (0.7%)	18 (0.7%)
Unknown	394 (6.0%)	147 (5.5%)
Number of primary cancer		
2	5594 (84.5%)	2368 (88.1%)
3	876 (13.2%)	277 (10.3%)
≥ 4	147 (2.2%)	42 (1.6%)
Age at 1^st^ cancer diagnosis (years)		
Range	0.1–89	13–84
Mean (*SD*)	48.4 (13.5)	50.2 (11.1)
Median (*IQR*)	49 (18)	50 (15)
Age at 2^nd^ cancer diagnosis (years)		
Range	11–89	19–89
Mean (*SD*)	58.7 (11.5)	61.0 (11.0)
Median (*IQR*)	59 (16)	61 (15)
Classification		
Positive in 21 genes*	833 (12.5%)	414 (15.4%)
*CHEK2* I157T	49 (0.7%)	26 (1.0%)
*MUTYH* carrier	110 (1.7%)	53 (2.0%)
Inconclusive	1541 (23.3%)	616 (22.9%)
Negative	4074 (61.6%)	1574 (58.6%)
ER and/or PR		
Yes (+ or +)	2863 (43.3%)	913 (34.0%)
No (− and − )	746 (11.3%)	257 (9.6%)
Unknown	3008 (45.5%)	1517 (56.5%)
HER2		
Yes	412 (6.2%)	135 (5.0%)
No	2192 (33.1%)	617 (23.0%)
Equivocal	12 (0.2%)	1 (0.04%)
Unknown	4001 (60.5%)	1934 (72.0%)
Triple Negative		
Yes	530 (8.0%)	175 (6.5%)
No	2995 (45.3%)	959 (35.7%)
Unknown	3092 (46.7%)	1553 (57.8%)
Bilateral breast cancer		
Yes	1007 (15.2%)	569 (21.2%)
No	5590 (84.5%)	2118 (78.8%)

*SD* standard deviation; *IQR* interquartile range; *ER* estrogen-receptor; *PR* progesterone-receptor; *HER*2 human epidermal growth factor receptor 2

^*^Includes MUTYH biallelic

**Fig. 2:**
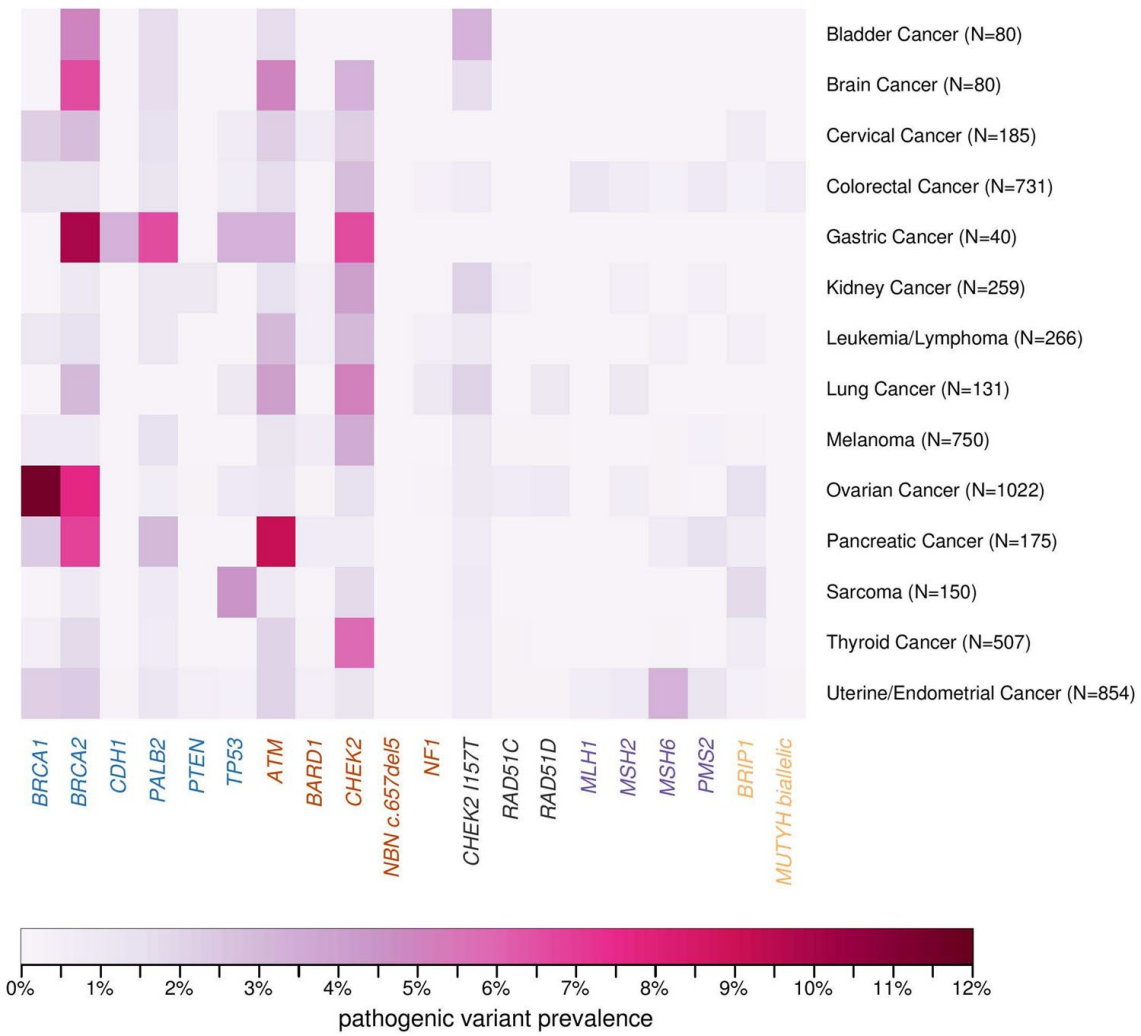
Heat map of cancer diagnosis among patients with multiple primary cancers and breast cancer

Overall, 14.1% (*n* = 932/6617) had one PV including *CHEK2* I157T and biallelic *MUTYH*, 23.3% (*n* = 1541) had no PV but at least one variant of uncertain significance (VUS), and 61.6% (*n* = 4074) had negative testing for all 21 genes of interest. The most frequently detected germline PVs were in *BRCA1* (2.3%, 95% *CI* 2.0–2.7%), *BRCA2* (2.3%, 95% *CI* 2.0–2.7%), *CHEK2* excluding I157T (2.0%, 95% *CI* 1.7–2.3%), *ATM* (1.5%, 95% *CI* 1.3–1.9%), and *PALB2* (0.9%, 95% *CI* 0.7–1.1%) (Fig. 2; Online Resource, Supplemental Table 1). An additional 0.7% (*n* = 49) had *CHEK2* I157T PV and an additional 1.7% (*n* = 110) had monoallelic *MUTYH*.

### Cancer combinations

We examined the frequency of PVs by the combination of cancer types among females with only two cancers (Fig. 2). As anticipated, among females with BC and OC (*n* = 1022), PVs were most frequent in *BRCA1* (8.6%, 95% *CI* 7.0–10.5%) and *BRCA2* (5.8%, 95% *CI* 4.5–7.4%). For breast and colorectal cancers (*n* = 731), PVs were most frequent in *CHEK2* excluding I157T (2.2%, 95% *CI* 1.4–3.5%), *ATM* (1.2%, 95% *CI* 0.6–2.4%), *MLH1* (0.8%, 95% *CI* 0.3–1.9%), and *PMS2* (0.7%, 95% *CI* 0.3–1.7%). Among 507 females with BC and thyroid cancer, *CHEK2* PVs (*n* = 22, 4.3%*,* 95% *CI* 2.9–6.5%) were detected most frequently. For the combination of BC and pancreatic cancer (*n* = 175), PVs were most frequent in *ATM* (6.9%, 95% *CI* 4.0–11.6%) followed by *BRCA2* (5.1%, 95% *CI* 2.5–9.8%). For the combination of BC and sarcoma (*n* = 150), PVs were most frequent in *TP53* (3.3%, 95% *CI* 1.2–8.0%) followed distantly by *BRIP1* and *CHEK2*. Among 80 females with BC and brain cancers, 11 had PVs of which PVs in *BRCA2* (*n* = 4, 5.0%, 95% *CI* 1.6–13.0%) occurred most frequently. Among 40 females with BC and gastric cancer, 10 had PVs: *BRCA2* (*n* = 3, 7.5%, 95% *CI* 2.0–21.5%) was the most frequent, followed by *PALB2* (*n* = 2, 5.0%, 95% *CI* 0.9–18.2%) and *CHEK2* (*n* = 2, 5.0%, 95% *CI* 0.9–18.2%). *ATM*, *CDH1*, and *TP53* PVs accounted for the 3 remaining females with BC and gastric cancer with PVs (for each: *n* = 1, 2.5%).

### PV frequency by order of breast cancer diagnosis

**Fig. 3:**
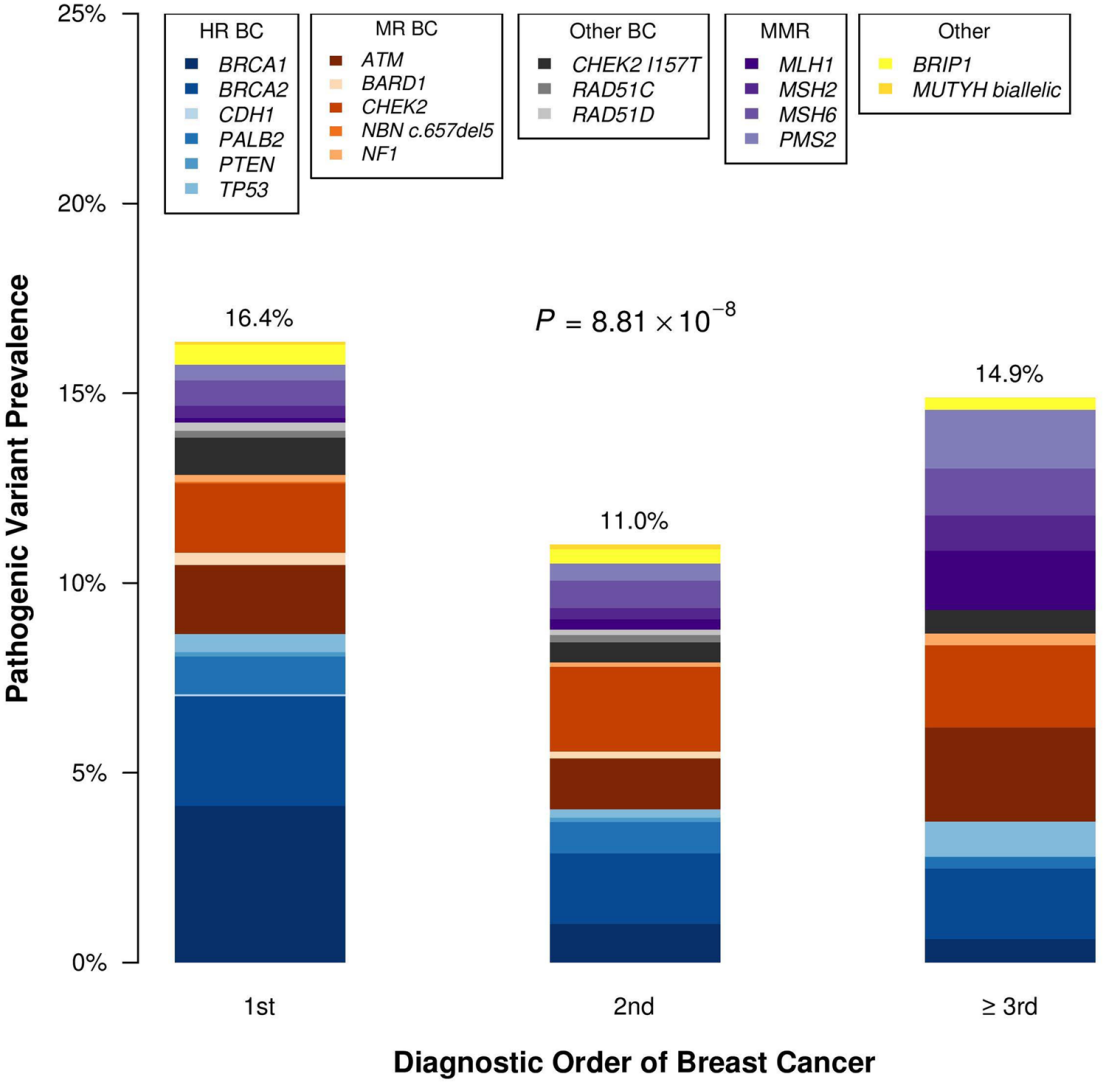
Prevalence of pathogenic variants among 21 selected genes by order of breast cancer diagnosis. Legend: HR BC, high-risk breast cancer genes; MR BC, moderate-risk breast cancer genes; Other BC, other breast cancer genes; MMR, mismatch repair; Other, non-breast cancer genes

Of the 6617 females in this study, 5651 had evaluable data on the order of their first BC diagnosis (1st, 2nd, or ≥ 3rd cancer diagnosis). For 966 females, the diagnostic order of their (first, if multiple) BC was ambiguous, and therefore, they excluded from this analysis. The frequency of PVs was 16.4% (95% *CI* 15–17.8%) when BC was diagnosed 1st, 11.0% (95% *CI* 9.9–12.3%) when diagnosed 2nd, and 14.9% (95% *CI* 11.4–19.2%) when it was the > 3rd cancer diagnosis (*p* < 0.001, Fig. 3). The median age of BC was younger when BC diagnosis was the 1st cancer diagnosis (50, *IQR* 15) compared to when BC was the 2nd cancer diagnosis (58, *IQR *17) or ≥ 3rd (64, *IQR* 17) cancer diagnosis. The frequency of PVs in high-risk BC genes (*BRCA1, BRCA2, CDH1, PALB2, PTEN*, and *TP53*) was greater for patients with BC as the 1st cancer diagnosis, 8.7% (95% *CI* 7.7–9.8%) compared to BC as a 2nd or ≥ 3rd cancer diagnosis, 4.1% (95% *CI* 3.4–4.9%) and 3.7% (95% *CI* 2.1–7.9%), respectively (*p* for trend < 0.001). The frequency of MMR PVs was highest for patients with BC as their ≥ 3rd cancer diagnosis, 5.3% (95% *CI* 3.3–8.3%) compared to 1st or 2nd cancer, 1.5% (95% *CI* 1.1–2.1%) and 1.7% (95% *CI* 1.3–2.3%), respectively (*p* for trend = 0.001). Females who were diagnosed with BC as their 2nd cancer had a younger median age at genetic testing (61, *IQR* 16) than those with BC as their 1st cancer diagnosis (65, *IQR* 14) (Online Resource, Supplemental Table 1).

### PV frequency by order ovarian cancer diagnosis

The order of OC diagnosis (1st, 2nd or ≥ 3rd cancer) was available for 1,247 of the 2,443 females with OC in this cohort. The frequency of PVs was 14.4% (95% *CI* 8.8–22.8%) when OC was the 1st cancer diagnosis, 20.3% (95% *CI* 18.0–22.8%) when diagnosed second 2nd and 22.8% (95% *CI* 14.9–33.2%) when it was the ≥ 3rd cancer diagnosis (*p* for trend = 0.15 Online Resource, Supplemental Fig. 1). The median age of OC was younger when the OC diagnosis was the 1st cancer diagnosis (46, *IQR* 19), compared to when OC was the 2nd cancer diagnosis (63, *IQR* 17) or ≥ 3rd cancer diagnosis (67, *IQR* 14.5) (Online Resource, Supplemental Table 2).

### Cancer interception

**Fig. 4:**
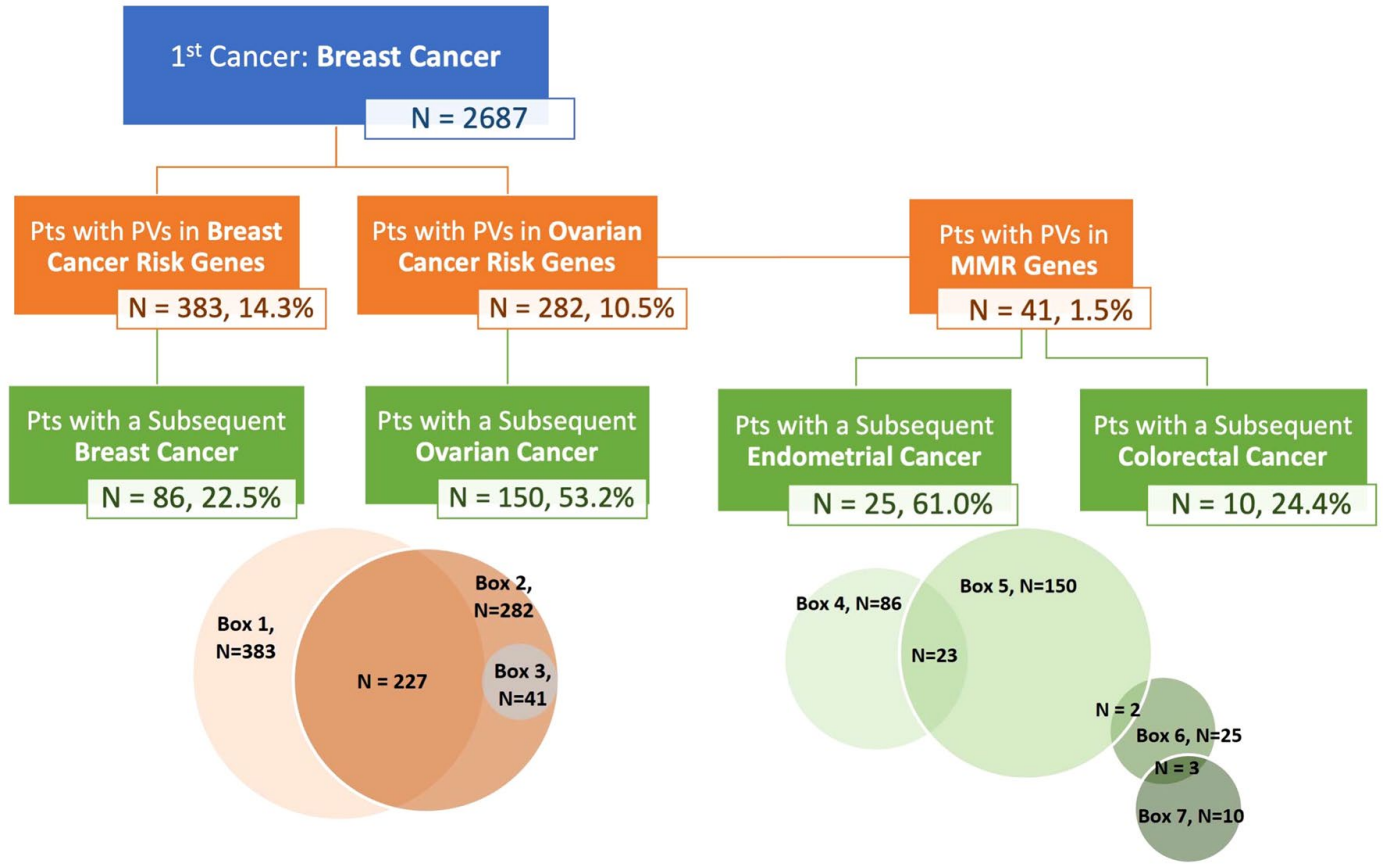
Subsequent genetic cancers among females first diagnosed with breast cancer. Legend: Pts, patients; PV(s), pathogenic or likely pathogenic variant(s). Top: Box 1 includes breast cancer genes (*ATM, BARD1, BRCA1/2, CDH1, CHEK2, NBN 657del, NF1, PALB2, PTEN, RAD51C/D*, and *TP53*). Box 2 includes ovarian cancer genes (*BRCA1/2, BRIP1, PALB2, RAD51C/D* and MMR genes. Box 3 includes mismatch repair genes (*MLH1, MSH2, MSH6,* and *PMS2).* Bottom: Venn diagrams display overlapped patients from above as follows: Boxes 1 and 2, *n* = 227; Boxes 2 and 3, *n* = 41; Boxes 4 and 5, *n* = 23; Boxes 5 and 6, *n* = 2; Boxes 6 and 7, *n* = 3. As we excluded patients with multiple mutations, there were no overlapped patients for Boxes 1 and 3

Among females with breast cancer as their first cancer diagnosis (*n* = 2687), 14.3% (*n* = 383) had a PV in a BC risk gene, 10.5% (*n* = 282) had a PV in an OC risk gene, and 1.5% (*n* = 41) with a PV in a MMR gene (Fig. 4). Among females with a PV in a BC risk gene (*ATM, BARD1, BRCA1, BRCA2, CDH1, CHEK2, NBN- 657del, NF1, PALB2, PTEN, RAD51C, RAD51D*, and *TP53*), 22.5% (86/383) had a subsequent BC diagnosis. Among females with a PV in an OC risk genes (*BRCA1, BRCA2, BRIP1, PABL2, RAD51C, RAD51D* and MMR genes), 53.2% (150/282) had a subsequent OC diagnosis. Among those with Lynch syndrome due to a PV in a MMR gene (*MLH1, MSH2, MSH6*, and *PMS2*), 61.0% (25/41) were subsequently diagnosed with endometrial cancer, 24.4% (10/41) with colorectal cancer, and 3 patients with both. In sum, 16.4% (440/2687) of patients with BC as their first cancer diagnosis were found to have a PV in a cancer susceptibility gene and this included two patients with biallelic *MUTYH*. Among these 438 females with an identified PV in a BC risk gene, OC risk gene, or MMR gene, 243 (55.5%) females had 271 subsequent diagnoses of breast, ovarian, endometrial, or colorectal cancer associated with their germline PV.

Among females diagnosed first with OC, 13.4% (13/97) had a PV in a BC risk gene or an MMR gene (Online Resource, Supplemental Fig. 2). Of the 10 patients who had a PV in a BC risk gene, 60.0% (*n* = 6) developed a subsequent BC.

## Discussion

In this selected cohort of females with breast cancer and a non-breast cancer diagnosis, 14.1% (932/6617) had a single PV in one of 21 hereditary cancer genes. Owing to early detection and targeted therapies, BC survivorship has improved, and identifying BC patients with germline cancer susceptibility provides an opportunity to enhance their care through cancer prevention or interception.

Our findings expand on prior work where the frequency of non-*BRCA1*/*BRCA2* PVs for women with MPCs (BC and another cancer diagnosis, including a second BC diagnosis) was 8.5% (47/551) among those followed in a high-risk BC program [2]. Herein, we report a similar rate of non-*BRCA1/BRCA2* PVs (7.9% [526/6617], 95% *CI* 7.3–8.6%), despite differences in inclusion criteria. One difference between the cohorts was that the prior study had more females with bilateral or contralateral BC (44.1%, 243/551) compared to our study (15.2%, 1007/6617).

Most patients did not undergo germline MGPT until well after their second cancer diagnosis. The median age at first cancer diagnosis was 49 and was 10 years prior to the median age of second cancer diagnosis at 59. The median age of MGPT was 63 in this cohort, ascertained from 2012 to 2016. This lengthy interval to germline genetic testing is consistent with prior work [9, 17, 18]. One study evaluating genetic test rates over an overlapping time period (from 2012 to 2019) found that only 25.2% of eligible patients with BC and 34.3% of females with OC received germline genetic testing, and many experienced barriers to testing [8]. Prior to 2012, MGPT was not widely available, and utilization of MGPT has increased since then for patients with BC and OC [8].

The greatest prevalence of PVs by cancer combination was among females with BC in combination with gastric cancer (25.0%), OC (22.3%), and pancreatic cancer (20.0%). *CHEK2* PVs were frequent in females with BC who also had either thyroid cancer or kidney cancer. This association has been previously described by our group and others [2, 19, 20]. The prevalence of PVs in *ATM* among females with BC and pancreatic cancer was greater than the prevalence of *BRCA2* PVs. This finding, while unexpected, is consistent with recent data delineating the age-specific penetrance of pancreatic cancer among individuals with PVs in *ATM*.[21]

The prevalence of PVs varied by the order of diagnosis of BC (1st: 16.4%, 2nd: 11.0%, and ≥ 3rd: 14.9%) and these differences were statistically significant. However, in all groups, the PV prevalence was > 10%, indicating that females with BC and another cancer diagnosis have a high rate of harboring a PV in a cancer susceptibility gene. As anticipated, females diagnosed with BC as their first cancer had a younger median age and were more likely to harbor a PV in a high-risk BC gene (*BRCA1, BRCA2, CDH1, PALB2, PTEN*, and *TP53*). Interestingly, their median age of genetic testing was older (65) than patients with BC as their second cancer diagnosis (61). This is likely due to secular trends, such as temporal changes in access to and greater utilization of genetic testing for females with BC.

Among the 2,687 patients with a first diagnosis of BC, 16.4% harbored a PV in a BC risk gene (*ATM, BARD1, BRCA1, BRCA2, CDH1, CHEK2, NBN- 657del, NF1, PALB2, PTEN, RAD51C, RAD51D*, and *TP53*), 10.5% in an OC risk gene ((*BRCA1, BRCA2, BRIP1, PABL2, RAD51C, RAD51D* and MMR genes)), and 1.5% in an DNA MMR gene (*MLH1, MSH2, MSH6*, and *PMS2*). There were 271 subsequent cancers identified in 243 of the 438 females with PVs that may have been intercepted through precision surveillance and/or prevention. These are all cancers for which there is enhanced surveillance or risk reduction based on the PV as recommended by the NCCN Guidelines® [16]. For females with PVs in *BRCA1, BRCA2, BRIP1, PABL2, RAD51C, RAD51D*, and MMR genes, bilateral salpingo-oophorectomies are the standard of care. For females with PVs in MMR genes, colorectal and uterine cancers can be prevented through frequent colonoscopies and hysterectomy, respectively. Interception of these cancers in females with germline PVs is associated with reduced morbidity and reduced mortality.

Our understanding of subsequent BCs and contralateral BCs is evolving, and data show that females with a PV in *BRCA1, BRCA2, PALB2, TP53*, or the *CHEK2* 1100del PV have a high risk of a subsequent and/or contralateral BCs in particular, if their first BC was diagnosed at a young age [1, 3, 22–24]. Moreover, about one-fifth of subsequent BCs (22.5%, 86/383) were in females with a PV in a BC risk gene.

In 2019, the American Society of Breast Surgeons released a consensus statement that germline genetic testing should be available for all patients with BC, although they acknowledged this approach may not be feasible [25]. In June 2021, when the OlympiA study showed a role for adjuvant olaparib for patients with high-risk Stage 2–3 BC and a germline PV in *BRCA1* or *BRCA2*,[7], the NCCN Guidelines® endorsed germline testing for any females with a new diagnosis of nonmetastatic BC who would be candidates for adjuvant PARP inhibitor therapy [26]. With the OlympiA results, there have been calls to expand testing to all patients with BC regardless of stage, tumor subtype, and clinical status [27, 28]. Experts have advocated for genetic testing to be listed on the WHO Essential Diagnostic List for patients with cancer regardless of race, ethnicity, ancestry, and geography [5]. Universal testing for all patients with cancer has been explored in research settings with many actionable results identified [29–31]. The President’s Cancer Panel recommends “an assessment of eligibility for germline genetic testing for all people diagnosed with cancer” [32] and calls grow for the implementation of germline genetic testing for all patients with solid organ cancers since the results impact clinical care [5, 29, 31].

Limitations of this study include the ascertainment biases inherent in a cohort selected for genetic testing for cancer predisposition. Our study population had to have survived their first cancer to be diagnosed with a subsequent cancer. Females with MPCs, including BC, may have been more likely to have germline genetic testing if diagnosed with their first cancer diagnosis at a younger age (median age of 48) or if they have a family history of cancer. The testing population is racially homogenesis (predominately White). In this cohort of females with BC, some may have had genetic testing with smaller panels at other laboratories (i.e., *BRCA1/BRCA2* only) prior to having testing for these 21 genes. Variant classification and the clinical impact of specific variants may be refined over time (e.g., *CHEK2* low-risk variants I157T, S428F, and T476M)[20]. Next, our analyses were limited to the 21 selected genes, and it is possible that if we evaluated more genes, such as *BAP1, CDKN2A, FH, MEN1, MEN2, MITF, POT1, RET, SDHx, TERT*, and *VHL,* additional cancers could be intercepted. For patients found to have genetic risk of melanoma, endocrine neoplasms, and/or renal cell carcinoma, there are efficacious guidelines for screening that should be utilized [33–35]. We have previously reported on the frequency of unexpected germline PVs among patients with BC [36, 37] and on intercepted genetic cancers [38]. Information on BC hormone receptor subtype was only available for some patients and therefore was not analyzed. Females with multiple PVs were removed from the analytic cohort and are the subject of a separate manuscript. While compelling, the subgroup analysis of opportunities for interception of subsequent cancers among females with PVs first diagnosed with BC or OC may be conservative. For example, we did not include females with multiple PVs, and females with *ATM* or *BRCA2* PVs who later developed pancreatic cancer, despite progress in earlier detection of pancreatic cancer [39–42]. While we provide a conservative estimate of opportunities for cancer interception, this is reasonable, as some patients may not have access to intensive care for a germline PV. Despite these limitations, a strength of this work is that, short of counterfactual modeling[43] or randomized trials of genetic testing, the opportunities for subsequent cancer interception would be indeterminable.

The high prevalence (> 10%) of PVs among patients with breast cancer and a non-breast cancer diagnosis supports germline MGPT of this population, irrespective of diagnostic order of BC, age at their cancer diagnoses, breast tumor subtype, BC stage, and type of non-BC. With expanding indications for germline genetic testing in breast oncology, including identifying females with nonmetastatic Stage II-III disease who are candidates for adjuvant olaparib, more PVs will be identified. The identification of PVs in females with BC not only informs their cancer treatment, but also their risk for subsequent cancers, and opportunities for interception of those cancers for themselves and for their families. Since outcomes are worse for patients with a subsequent cancer diagnosis compared to patients with an initial cancer diagnosis for most cancer types [44], opportunities for reducing subsequent cancer morbidity and mortality in females with breast cancer and pathogenic variants are increasingly important.

## Supplementary Information

Below is the link to the electronic supplementary material.

Supplementary file1 (DOCX 375 KB)

## Data Availability

All data generated or analyzed during this study are included in this published article.
