# Correction to: Pathogenic variants among females with breast cancer and a non‑breast cancer reveal opportunities for cancer interception

**DOI:** 10.1007/s10549-023-06928-w

**Published:** 2023-05-13

**Authors:** Brittany L. Bychkovsky, Min-Tzu Lo, Amal Yussuf, Carrie Horton, Parichehr Hemyari, Holly LaDuca, Judy E. Garber, Rochelle Scheib, Huma Q. Rana

**Affiliations:** 1 Department of Medical Oncology, Dana-Farber Cancer Institute, 450 Brookline Ave, Boston, MA 02215 USA; 2 Breast Oncology Program, Dana-Farber Brigham Cancer Center, Boston, MA USA; 3 Division of Cancer Genetics and Prevention, Dana-Farber Cancer Institute, Boston, MA USA; 4 Harvard Medical School, Boston, MA USA; 5 Ambry Genetics, Aliso Viejo, CA USA

**Correction to: Breast Cancer Research, Treatment** 10.1007/s10549-023-06870-x

In the original publication of the article, the reference citations in the text have been incorrectly processed. The original article has been corrected.

