# Supplementary material for: Pathogenic variants among females with breast cancer and a non-breast cancer reveal opportunities for cancer interception
Source: Breast Cancer Res Treat. 2023 Mar 1;200(1):63–72. doi: 10.1007/s10549-023-06870-x (PMC10224857; doi:10.1007/s10549-023-06870-x)
Supplement: Supplementary file 1 — Supplementary file1 (DOCX 375 KB) [file 10549_2023_6870_MOESM1_ESM.docx]

**Online Resources:**

**Supplemental Tables and Figures**

**Article: Pathogenic variants among females with breast cancer and a non-breast cancer reveal opportunities for cancer interception**

**Journal:** Breast Cancer Research and Treatment

**Authors:** Brittany L. Bychkovsky, MD, MSc; Min-Tzu Lo, PhD; Amal Yussuf, BS; Carrie Horton, MS; Parichehr Hemyari, PhD; Holly LaDuca, MS; Judy E. Garber, MD, MPH; Rochelle Scheib, MD, MPH; Huma Q. Rana, MD, MPH

**Corresponding Author:**

Brittany L. Bychkovsky

Dana-Farber Cancer Institute

450 Brookline Ave

Boston, MA 02215

Phone: 617-632-3800

Email: Brittany_Bychkovsky@dfci.harvard.edu

**Supplemental Table 1**

| **Diagnostic order of first breast cancer** | **Number (%)** | **Median Age of Breast Cancer (IQR)** | **Median Age at Testing (IQR)** | **N of PVs** | **PV Prevalence**  **(95% CI)** | **p value for trend** |
| --- | --- | --- | --- | --- | --- | --- |
| 1st | 2687 (47.6%) | 50 (15) | 65 (14) | 440 | 16.4% (15.0%, 17.8%) | <0.001 |
| 2nd | 2641 (46.7%) | 58 (17) | 61 (16) | 291 | 11.0% (9.9%, 12.3%) |  |
| ≥ 3rd | 323 (5.7%) | 64 (17) | 66 (16) | 48 | 14.9% (11.4%, 19.2%) |  |

IQR, interquartile range; PV(s), pathogenic or likely pathogenic variant(s); CI, confidence interval

**Supplemental Fig. 1**


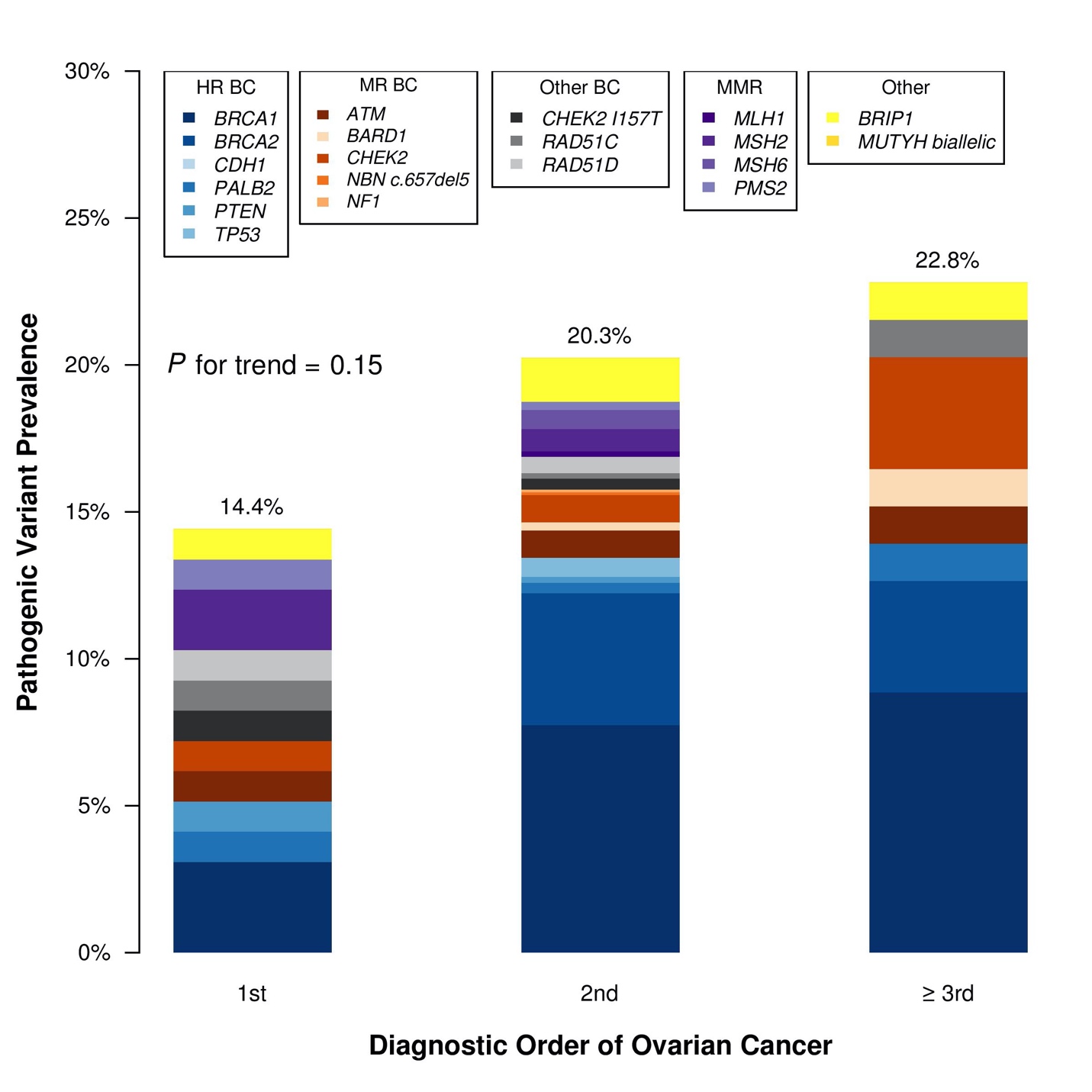


HR BC, high-risk breast cancer genes; MR BC, moderate-risk breast cancer genes; Other BC, other breast cancer genes; MMR, mismatch repair; Other, non-breast cancer genes.

**Supplemental Table 2**

| **Diagnostic order of first ovarian cancer*** | **Number (%)** | **Median Age of Breast Cancer (IQR)** | **Median Age at Testing (IQR)** | **N of PVs** | **PV Prevalence**  **(95% CI)** | **p value for trend** |
| --- | --- | --- | --- | --- | --- | --- |
| 1st | 97 (7.8%) | 46 (19) | 65 (15) | 14 | 14.4% (8.8%, 22.8%) | 0.15 |
| 2nd | 1071 (85.9%) | 63 (17) | 66 (15) | 217 | 20.3% (18.0%, 22.8%) |  |
| ≥ 3rd | 79 (6.3%) | 67 (14.5) | 68 (12.5) | 18 | 22.8% (14.9%, 33.2%) |  |

IQR, interquartile range; PV(s), pathogenic or likely pathogenic variant(s); CI, confidence interval

*There were a total of 2443 patients in the cohort with ovarian cancer, however, information on diagnostic order was only available for 1247 females.

**Supplemental Fig. 2**


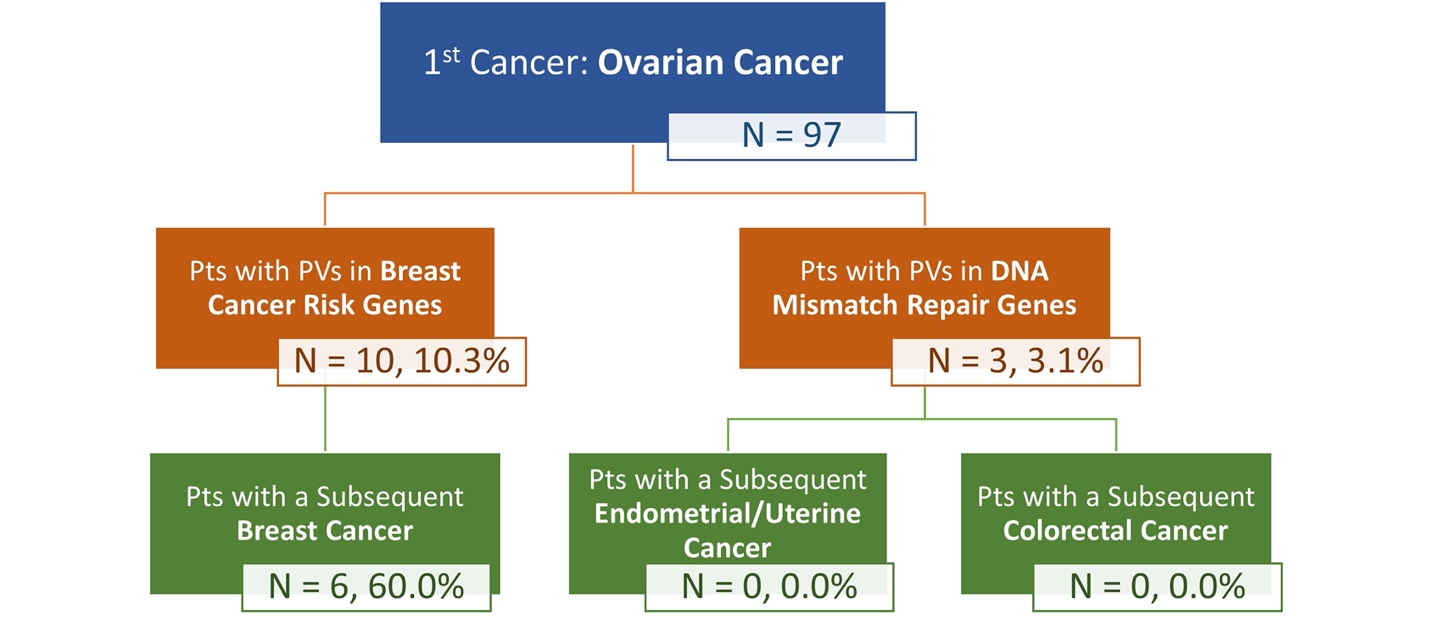


Pts, patients; PV(s), pathogenic or likely pathogenic variant(s)
